# Cross‐Reactive Neutralizing Antibodies Between ZIKV and DENV May Exhibit Serotype Specificity

**DOI:** 10.1002/biot.70323

**Published:** 2026-10-26

**Authors:** Lucimeire Antonelli da Silveira, Djairo Pastor Saavedra, Isabela Cinquini Junqueira, Jefferson do Carmo Dietz, Cristina Rodrigues de Oliveira, Leonardo Lopes‐Luz, Lorena Carvalho de Souza Chaves, Bergmann Morais Ribeiro

**Affiliations:** ^1^ Department of Biosciences and Technology Institute of Tropical Pathology and Public Health Federal University of Goiás Goiânia Goiás Brazil; ^2^ Innovation Hub in Point of Care Technologies Federal University of Goiás‐Merck S/A Alliance Goiânia Goiás Brazil; ^3^ Department of Biomedical Engineering Emory University Atlanta Geogia USA; ^4^ Department of Cell Biology Institute of Biology University of Brasília Brasília Brazil

**Keywords:** antibodies, dengue virus, flavivirus, neutralizing antibodies, Zika virus

## Abstract

Dengue virus (DENV) and Zika virus (ZIKV) are public health threats, causing substantial morbidity and mortality worldwide. The co‐circulation of DENV and ZIKV complicates prevention and control efforts, highlighting the importance of studying cross‐reactive antibodies, vaccines, and immune modulators. This study explores the cross‐reactivity and neutralizing capacity of antibodies against DENV elicited by immunization with a ZIKV protein extract. Female isogenic BALB/c mice were immunized with ZIKV protein extract derived from C6/36 cell cultures. Splenic B lymphocytes from immunized mice were fused with myeloma cells using polyethylene glycol (PEG) to generate hybridomas. The resulting antibodies were assessed using ELISA and plaque reduction neutralization tests (PRNT) against ZIKV and DENV including their structural and nonstructural proteins. Our findings showed that immunization induced a diverse antibody response, including antibodies recognizing epitopes unique to ZIKV as well as epitopes shared with DENV. Notably, neutralizing antibodies targeting conserved epitopes, particularly those shared with DENV‐1, were also identified against other DENV serotypes. These results suggest the presence of cross‐reactive neutralizing antibody responses between ZIKV and DENV, potentially exhibiting serotype‐specific reactivity. Collectively, this study provides experimental evidence of flavivirus cross‐reactivity and contributes to a more refined understanding of humoral responses in settings where these viruses co‐circulate.

## Graphical Abstract and Lay Summary

Immunization with ZIKV protein extract may induce serotype‐specific neutralizing antibodies against DENV.

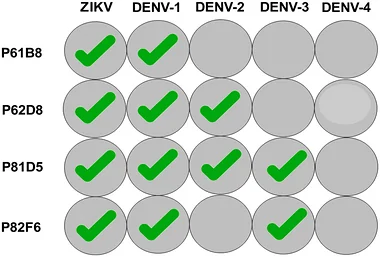

## 1 Introduction

Dengue virus (DENV) and Zika virus (ZIKV) are significant public health concerns responsible for widespread morbidity and mortality worldwide. Both belong to the Orthoflavivirus genus (Flaviviridae family) and are transmitted by Aedes spp. mosquitoes [1]. These enveloped viruses share a positive‐sense, single‐stranded RNA genome encoding ten proteins: capsid (C), precursor membrane (prM), envelope (E), and five nonstructural proteins (NS1–NS5). DENV and ZIKV share approximately 55%–58% genomic sequence similarity, often leading to cross‐reactive immune responses [1].

DENV is estimated to cause 400 million infections annually, primarily in tropical and subtropical regions [2]. While most cases are asymptomatic or mild, severe presentations, including hemorrhagic fever and shock, can occur [1]. Similarly, ZIKV infections are frequently asymptomatic or mild but are associated with severe complications, including Guillain‐Barré syndrome in adults and congenital abnormalities such as microcephaly in infants born to infected mothers [3].

The geographic co‐circulation of DENV and ZIKV, exemplified by outbreaks in the Pacific Islands (2013 and 2014) and Brazil (2015), highlights the challenges of managing these infections [4]. Accurate diagnostic tools and effective therapeutic options are urgently needed to distinguish and treat these closely related viruses. In this context, the Antibody Production Center of the Midwest (CEPRACO) at the Federal University of Goiás initiated the production of anti‐ZIKV murine monoclonal and polyclonal antibodies in 2017, aiming to support flavivirus research.

The phenomenon of cross‐reactivity between flaviviruses, while complicating diagnosis, presents opportunities for immunotherapy [1]. Monoclonal antibody (mAb) therapy, widely adopted since the 1980s, addresses several limitations of conventional treatments, such as drug toxicity, resistance, and limited efficacy [5]. However, vaccines like Dengvaxia, approved for DENV in Brazil, have revealed challenges associated with antibody‐dependent enhancement (ADE), where prior DENV infections may exacerbate disease severity upon reinfection [6]. Newer vaccines, such as Qdenga, offer improvements by not requiring serological testing [7].

This study investigates the cross‐reactive potential and neutralizing capacity of anti‐ ZIKV antibodies against both ZIKV and DENV. By analyzing in vitro‐produced murine antibodies from ZIKV protein extract‐immunized animals, we aim to contribute to the understanding of immune responses to flaviviruses and their potential relevance to vaccine responses in regions where these viruses co‐circulate, while also providing preliminary insights into the potential applicability of immunotherapy.

## 2 Materials and Methods

### 2.1 Preparation of ZIKV and DENV Protein Extracts

To obtain ZIKV (H/PF/2013) and DENV‐1 (strain Hawaii), DENV‐2 (strain N. Guinea C), DENV‐3 (strain H 87), and DENV‐4 (strain BC 295/97) protein extracts, *Aedes albopictus* C6/36 cells cultured in vitro were infected with the respective flaviviruses. The entire culture supernatant was removed, and an aliquot of viral suspension at a concentration of approximately 0.1–0.2 MOI was added. The culture was incubated for 1 h at 25°C–28°C with gentle mixing every 20 min to promote viral adsorption. After this stage, Leibovitz medium (Sigma–Aldrich) was added, and the cell suspension was incubated at 25°C–28°C for approximately 7 to 10 days for both ZIKV and DENV. When the proportion of cells showing cytopathic effect exceeded 80%, the culture flasks were frozen at ‐80°C. After 24 h, the material was thawed and centrifuged at 3000 rpm for 10 min. The supernatant containing the viruses was filtered through 0.45 µm filters (Millipore), and the cell pellet was discarded. Subsequently, the virus was concentrated using polyethylene glycol (PEG 3100; Sigma) mixed with the viral suspension at a 1:4 (v/v) ratio.

### 2.2 Production of Antibodies

The murine polyclonal and monoclonal antibodies used in this study were produced in vitro at the Antibody Production Center of the Midwest (CEPRACO) at the Federal University of Goiás using hybridoma technology (St Groth, Scheidegger, 1980). Hybridomas were generated by fusing B lymphocytes from the spleen of female isogenic Balb/c mice immunized with ZIKV protein extract and SP2 myeloma cells. Clones were separated by limiting dilution. The cell culture supernatants were concentrated using a combined filtration and centrifugation system (3000 rpm for 30 min at 4°C) in a tube with a 10,000‐dalton regenerated membrane (Millipore), and the antibodies were purified with affinity chromatography columns (Cytiva) on an ÄKTA Purifier system (GE Healthcare). For neutralization assays, murine antibodies were dialyzed in phosphate‐buffered saline (PBS), quantified using the bicinchoninic acid method (Pierce BCA Protein Assay Kit), and filtered using sterile syringe filters with a 0.22 µm PVDF membrane (Millipore).

### 2.3 Enzyme‐Linked Immunosorbent Assay (ELISA)

Each 96‐well microplate (Corning Costar) was coated with ZIKV protein extract diluted 1:200 in carbonate buffer at 4°C–8°C for 18–24 h. The supernatant was then discarded, and wells were washed three times with 200 µL of phosphate‐buffered saline (PBS pH 7.2) with 0.05% Tween 20. Nonspecific binding sites were blocked for 2–3 h at 37°C with 100 µL of PBS with 0.05% Tween 20 and 5% nonfat powdered milk (Nestlé Molico). The supernatant was then discarded, and wells were washed three times with 200 µL of PBS with 0.05% Tween 20.

In the assays, sera from immunized mice were used as positive controls and from nonimmunized animals as negative controls, diluted 1:100 in PBS with 0.05% Tween 20 and 0.5% nonfat powdered milk, and hybridoma culture supernatants in 50 µL volumes were incubated for 1 h at 37°C. Wells were subsequently washed three times with 200 µL of PBS with 0.05% Tween 20, and 50 µL of anti‐mouse IgG conjugate (Sigma–Aldrich) diluted 1:4000 in PBS with 0.05% Tween 20 and 0.5% non‐fat powdered milk was added. Incubation was carried out for 1 h at 37°C, and the wells were washed three times with 200 µL of PBS with 0.05% Tween 20.

Next, 50 µL of tetramethylbenzidine chromogenic solution was added, and the plate was incubated for 10 min at room temperature, protected from light. Finally, 50 µL of stop solution (2 N HCl) was added, and readings were taken on a plate reader (Thermo–Fisher Scientific) at 450 nm.

### 2.4 Capture Enzyme‐Linked Immunosorbent Assay (Capture ELISA)

Capture assays were performed to confirm the specific reactivity of the antibodies produced by the hybridomas. 96‐well microplates (Corning Costar) were coated with purified antibodies (Poly 1–10) at a concentration of 20 µg/ml in carbonate buffer at 4°C–8°C for 18–24 h. A commercial anti‐flavivirus antibody (D14G2415, ATCC HB112) was used as a positive control in this assay. The supernatant was then discarded, and wells were washed three times with 200 µL of phosphate‐buffered saline (PBS, pH 7.2) with 0.05% Tween 20. Nonspecific binding sites were blocked for 2–3 h at 37°C with 100 µL of PBS with 0.05% Tween 20 and 5% nonfat powdered milk (Nestlé Molico). The supernatant was then discarded, and wells were washed three times with 200 µL of PBS with 0.05% Tween 20. In the assays, A pooled serum sample from individuals with confirmed Zika virus infection was used at a 1:50 dilution in carbonate buffer, followed by incubation of the reaction for 1 h at 37°C. Wells were subsequently washed three times with 200 µL of PBS with 0.05% Tween 20, and 50 µL of anti‐human IgG conjugate (Sigma–Aldrich) diluted 1:500 in PBS with 0.05% Tween 20 and 0.5% nonfat powdered milk was added. Incubation was carried out for 1 h at 37°C, and the wells were washed again three times with 200 µL of PBS with 0.05% Tween 20. Next, 50 µL of tetramethylbenzidine chromogenic solution was added, and the plate was incubated for 10 min at room temperature, protected from light. Finally, 50 µL of stop solution (2 N HCl) was added, and readings were taken on a plate reader (Thermo–Fisher Scientific) at 450 nm.

### 2.5 Plaque Reduction Neutralization Test (PRNT)

The neutralizing capacity of the antibodies against ZIKV and DENV was determined using the Plaque Reduction Neutralization Test (PRNT), adapted from Stefano et al. (1999) [8]. Vero cells were cultured in 24‐well plates (3.0 × 10^5^ cells/well) in MEM medium (Gibco) supplemented with 10% fetal bovine serum (Gibco) for 48–72 h at 37°C and 5% CO2. Antibodies were serially diluted in MEM medium, with concentrations ranging from 1000 µg/mL to 3.9 µg/mL, and mixed with an equal volume of culture medium containing viral particles (diluted 10^−4^, according to prior viral titration). After 30 min of virus‐antibody incubation at 37°C and 5% CO2, the mixtures were transferred in duplicate to plates containing Vero cells. A positive control with virus alone, without antibody, was included. The plates were incubated for 1 h under the same conditions, with gentle mixing every 20 min to ensure proper viral adsorption. Following this procedure, the virus‐antibody mixtures were removed, and cells were overlaid with 3% (w/v) carboxymethylcellulose supplemented with 10% 10X MEM, 10% penicillin‐streptomycin, and 10% fetal bovine serum. The cultures were maintained for approximately 7 to 10 days for ZIKV and DENV serotypes. To count cytopathic effects, the culture medium was discarded, and the plates were incubated for 18–24 h at room temperature with 40% formaldehyde and stained with crystal violet for 15 min. Viral control was used as a reference to calculate the percentage of neutralization according to the formula below.

**Neutralization rate (%)**

$$\begin{array}{cc} & \mathrm{Neutralization}\;\mathrm{rate}\;(\%)\\ & =\left(1-\frac{(\hat{\mathrm{CEs}})\;\text{of wells containing antibodies and virus}}{(\hat{\mathrm{CEs}})\;\text{of wells containing virus only}}\right)\times 100\% \end{array}$$

where (CEs) refers to the mean number of cytopathic effects for each well of the plate. Antibody concentrations with a neutralization rate greater than 50% were considered neutralizing.

**Variation between replicates (%)**

$$\begin{array}{cc} & \mathrm{Variation}\;\mathrm{between}\;\mathrm{replicates}\;(\%)\\ & =\frac{\text{Highest CE value}-\text{Lowest CE value}}{\text{Highest CE value}}\times 100\% \end{array}$$

where CE refers to the number of cytopathic effects for each well of the plate.

### 2.6 Preparation of Recombinant ZIKV and DENV Proteins

The production of the recombinant proteins Multi‐ZIKV and Multi‐DENV was previously described [9]. Recombinant proteins containing a region of the NS1 protein from DENV‐1, DENV‐2, and ZIKV, which includes multiple antigenic determinants between amino acid residues 57 and 126 [10], were fused to the polyhedrin protein of the baculovirus *Autographa californica* multiple nucleopolyhedrovirus (AcMNPV) [11, 12] (Figure S1). On the other hand, the recombinant E2 and E6 proteins (Figure S2) are derived from the expression of ZIKV structural proteins in insect cells. The E2 protein corresponds to the genomic region of the ZIKV polyprotein between positions 108–2489 (C, preM, and E), while E6 corresponds to positions 413–2489 (preM and E) [13].

### 2.7 Recognition of Recombinant ZIKV and DENV Proteins by Western Blotting

The ZIKV protein extract and recombinant proteins were separated by sodium dodecyl sulfate‐polyacrylamide gel electrophoresis (SDS–PAGE) using a 4% stacking gel and a 12% separating gel. The electrophoresis of samples and molecular weight markers (Bio‐Rad) was performed at 100 V for the first 20 min, followed by 1 h at 160 V. The proteins were then transferred to nitrocellulose membranes using a transblot apparatus (Bio‐Rad) for 30 min at 15 V. Membranes were blocked overnight at 2 to 8°C with 2% nonfat milk. After washing with PBS‐Tween 0.1%, membranes were incubated for 1 h with the following antibodies at a 1:500 dilution. After another washing step, membranes were incubated for 1 h with rabbit anti‐mouse IgG conjugated with alkaline phosphatase (Sigma–Aldrich). Finally, membranes were washed and incubated for 30 min with a detection system using NBT and BCIP.

### 2.8 Ethical Considerations

Female isogenic Balb/c mice used for the production of hybridomas secreting the antibodies tested in this study were bred and maintained at the Multiuser Center for Animal Production and Experimentation of the Institute of Tropical Pathology and Public Health at the Federal University of Goiás, in compliance with the principles and guidelines of the National Council for the Control of Animal Experimentation. The entire research protocol was submitted and approved in advance by the Ethics Committee on Animal Use of the Federal University of Goiás under approval number 101/2015.

## 3 Results

Female isogenic Balb/c mice (N = 5) were immunized with a protein extract derived from C6/36 mosquito cell cultures infected with ZIKV. The immunization protocol consisted of three doses administered intraperitoneally, each containing 10 µg of antigen formulated with 180 µg of aluminum hydroxide adjuvant per mouse. To validate the immunization strategy, serum samples were collected from the animals, and antibody production was confirmed using enzyme‐linked immunosorbent assay (ELISA). Following immunization, mouse spleens were harvested approximately 5 days later for the isolation of B lymphocytes, which were then immortalized using polyethylene glycol (PEG) to generate hybridomas, following the protocol described by St Groth and Scheidegger (1980) [14]. A total of 96 hybridomas were generated, expanded, and organized into 10 hybridoma groups (Poly 1–10). The antibody production capacity of these hybridomas was monitored for over 12 months and remained stable even after cell cryopreservation and thawing, as well as across different batches of produced antigens, older and freshly prepared antigen batches.

Antibodies were produced from hybridomas both before and after cloning via the limiting dilution technique. Prior to cloning, the antibodies were considered polyclonal, potentially recognizing multiple epitopes, while after cloning, they were classified as monoclonal, targeting specific epitopes.

Thus, the specificity of the antibodies produced by the hybridomas (Poly 1–10) was determined using different assays: indirect ELISA, capture ELISA, Western blot, and PRNT.

OD values obtained in ELISA assays from antibodies produced by these hybridomas ranged from 0.3 to 1.8 (positive control = 2.0 and negative control = 0.10).

Results from the capture ELISA assay confirmed the specificity of the produced antibodies (Poly1 ‐10), with OD values ranging from 1.44 to 2.21. An OD value of 3.02 was obtained for the commercially available anti‐flavivirus antibody (D14G2415, ATCC HB112), while values of 1.85 and 2.21 were observed for Poly 6 and Poly 8, respectively. In this assay, a pool of sera from uninfected individuals for both ZIKV and DENV was used as the negative control (OD = 0.26). To evaluate antibody interactions with components of C6/36 cell protein extracts, ELISA assays using ZIKV produced in Vero cells, diluted from 1:2 to 1:16, were also performed for these polyclonal antibodies and showed similar results, with OD values of 2.12 for poly 6 and 1.98 for poly 8.

The Poly 6 and Poly 8 hybridomas were selected and cloned for subsequent assays due to their sustained stability in antibody production over several months (>12) and their ability to neutralize ZIKV at lower concentrations (Tables 1 and 2). As shown in Table 1, antibodies derived from hybridomas Poly 6 and 8, along with their respective monoclonal clones, demonstrated discrimination between ZIKV and DENV protein extracts. These antibodies produced OD values at least two‐ to four‐fold higher for ZIKV protein extracts in ELISA assays, indicating their specificity, although cross‐reactivity with DENV protein extracts was also observed.

**TABLE 1:** ELISA with antibodies derived from hybridomas induced‐ZIKV protein extract with cross‐reactivity to DENV protein extract.

	ELISA (optical density)	
Antibodies	ZIKV	DENV 1‐4
Poly 6	1,5 (CO = 0,09; CP = 1,85)	0,3 (CO = 0,07; CP = 1,91)
P61B8	1,35 (CO = 0,13; CP = 2,0)	0,32 (CO = 0,10; CP = 2,2)
P61C3	1,49 (CO = 0,13; CP = 2,0)	0,64 (CO = 0,10; CP = 2,2)
P61D3	1,49 (CO = 0,13; CP = 2,0)	0,59 (CO = 0,10; CP = 2,2)
P61F8	1,55 (CO = 0,13; CP = 2,0)	0,62 (CO = 0,10; CP = 2,2)
P62D8	1,49 (CO = 0,13; CP = 2,0)	0,61 (CO = 0,10; CP = 2,2)
P62C11	1,29 (CO = 0,13; CP = 2,0)	0,36 (CO = 0,10; CP = 2,2)
Poly 8	2,1 (CO = 0,09; CP = 2,0)	0,5 (CO = 0,07; CP = 2,2)
P81B5	1,86 (CO = 0,13; CP = 2,0)	0,88 (CO = 0,10; CP = 2,2)
P81C9	1,84 (CO = 0,13; CP = 2,0)	0,87 (CO = 0,05; CP = 2,2)
P81D5	1,91 (CO = 0,13; CP = 2,0)	0,93 (CO = 0,05; CP = 2,2)
P81G9	1,88 (CO = 0,13; CP = 2,0)	0,97 (CO = 0,05; CP = 2,2)
P82F6	1,90 (CO = 0,13; CP = 2,0)	0,96 (CO = 0,10; CP = 2,2)

*Note*: CO, cut off; CP, positive control (sera from mices immunized with ZIKV).

**TABLE 2:** Percentage of ZIKV neutralization by murine antibodies Poly 1–10.

	Concentrations (µg/mL)–% neutralization								
Antibodies	1000	500	250	125	62.5	31.2	15.6	7.8	3.9
Poly 1	37%	52%	53%	40%	30%	41%	46%	32%	—
Poly 2	23%	32%	43%	39%	32%	36%	21%	18%	30%
Poly 3	72%	64%	56%	45%	44%	43%	43%	37%	44%
Poly 4	0%	28%	28%	29%	37%	44%	37%	47%	60%
Poly 5	44%	48%	41%	45%	40%	58%	32%	50%	48%
**Poly 6**	**87%**	**65%**	**53%**	**50%**	**62%**	**37%**	**34%**	**32%**	**46%**
Poly 7	60%	44%	39%	31%	21%	39%	31%	43%	21%
**Poly 8**	**68%**	**55%**	**57%**	**57%**	**42%**	**47%**	**48%**	**48%**	**46%**
Poly 9	50%	37%	46%	46%	48%	43%	47%	54%	—
Poly 10	38%	33%	37%	40%	40%	39%	32%	25%	—

*Note*: Bold values highlight the cloned polyclonal antibodies and PRNT performed against DENV serotypes.

Murine polyclonal and monoclonal antibodies were evaluated for their ability to neutralize ZIKV and DENV using the PRNT assay. Table 2 below summarizes the percentage of Zika virus neutralization by polyclonal antibodies (designated Poly 1–10) and monoclonal antibodies derived from cloned hybridomas Poly 6 and 8, tested across a range of concentrations from 1000 µg/mL to 3.9 µg/mL. As shown in Table 2, antibodies derived from hybridomas generated from B lymphocytes of ZIKV‐immunized mice exhibited variable neutralization capacities in vitro. Among these, the antibodies Poly 3, 6, 7, and 8 showed more pronounced neutralization activity. Hybridomas Poly 6 and 8 were selected for cloning due to their superior neutralization at concentrations between 1000–125 µg/mL, and their monoclonal clones largely retained this functional activity. Notably, clones P61B8 and P62D8 derived from Poly 6, as well as clones P81B5, P81D5, and P82F6 from Poly 8, exhibited neutralizing activity, as detailed in Table 3 and illustrated in Figures 1 and 2. These results raise the possibility that these clones may warrant further characterization and could potentially have therapeutic applications.

**TABLE 3:** Percentage of ZIKV neutralization by murine antibodies Poly 6,8 and mAbs

Anticorpos anti‐ZKV	500 µg/ml	250 µg/ml	125 µg/ml
Policlonal 6	65%	53%	50%
zP61B8	63%	50%	36%
P61F8	40%	40%	36%
P61C3	56%	40%	36%
P62D8	58%	54%	50%
P61D3	50%	43%	32%
P61C11	—	58%	50%
Policlonal 8	60%	55%	57%
P81H6	51%	48%	40%
P82F6	59%	51%	44%
P81D5	77%	59%	44%
P81C9	37%	31%	18%
P81G9	40%	37%	34%
P81E9	43%	40%	37%
P81B5	58%	55%	50%

**FIGURE 1:**
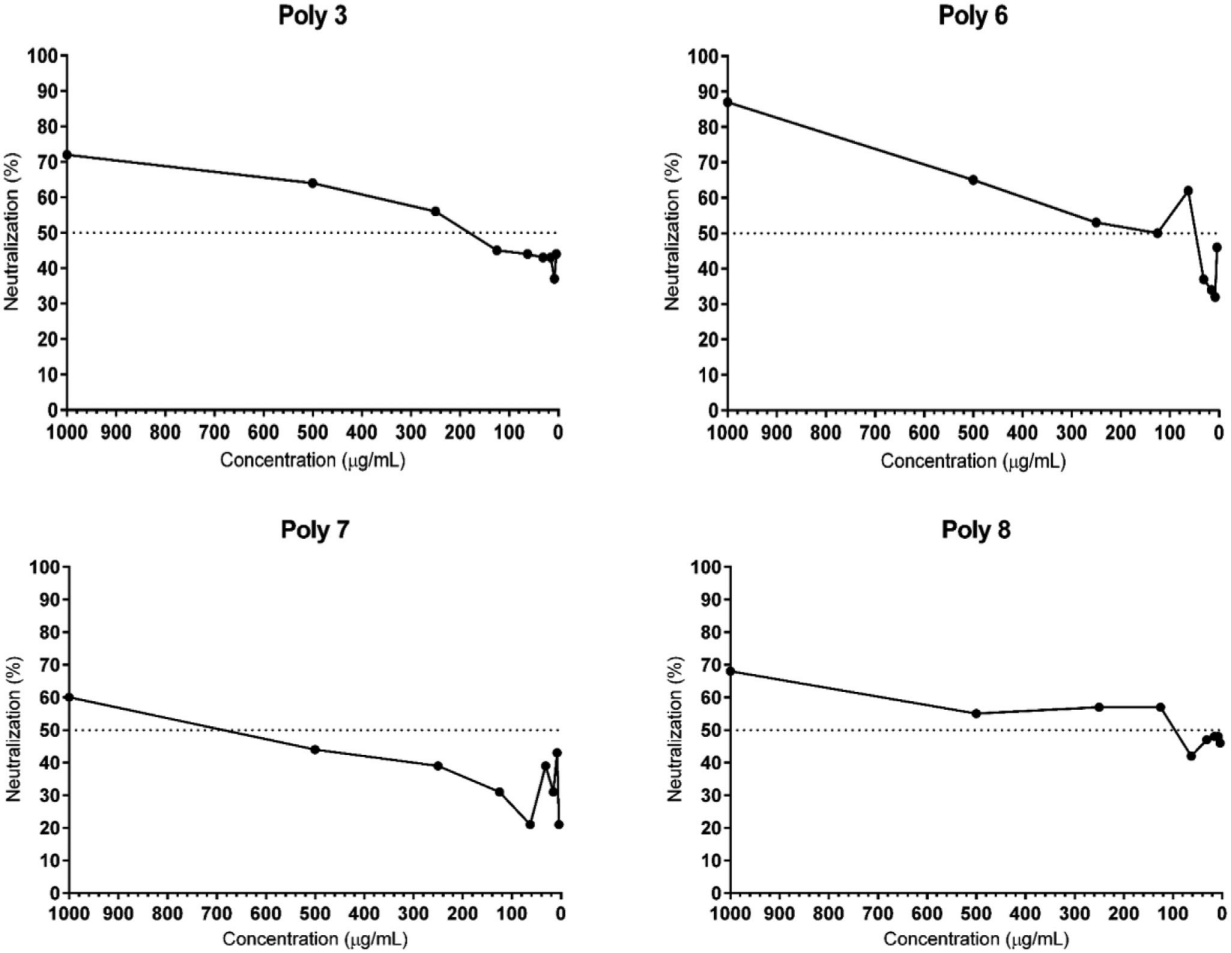
Neutralizing murine polyclonal antibodies 3,6,7,8 induced in response to ZIKV

**FIGURE 2:**
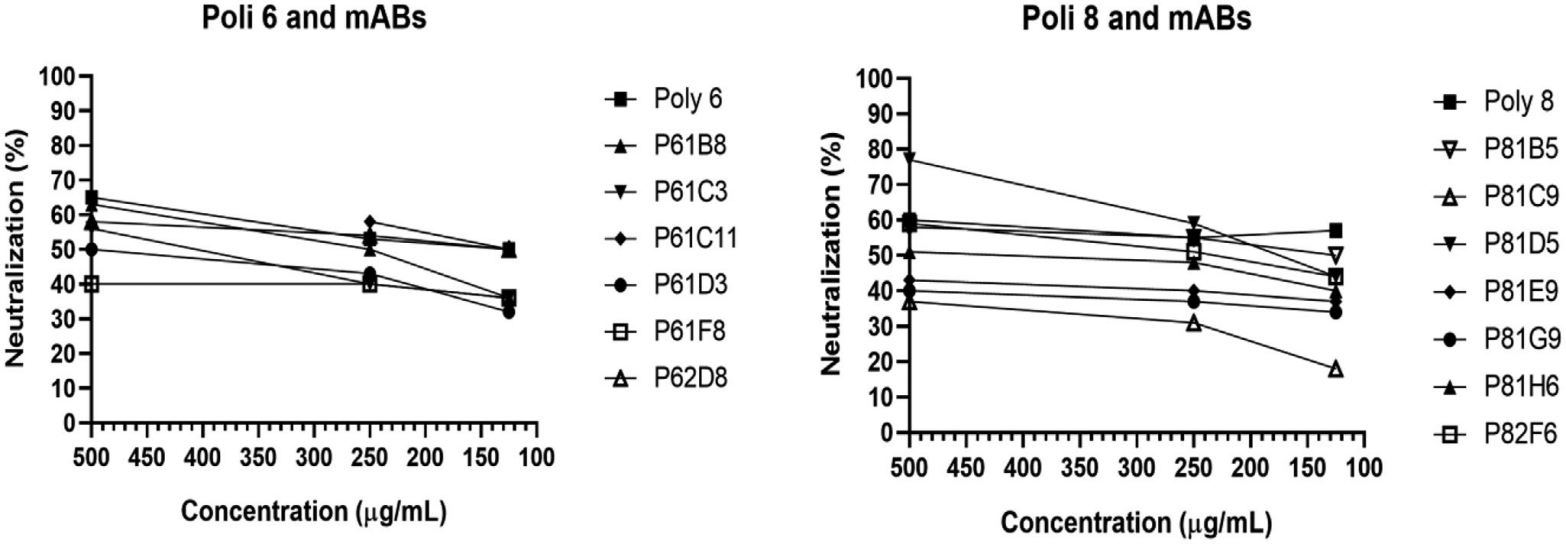
Neutralizing activity of murine polyclonal 6,8 and monoclonal antibodies produced in response to ZIKV protein.

The monoclonal antibodies exhibiting the highest ZIKV neutralization capacity were further evaluated for their ability to neutralize DENV, as shown in Figures 3 and 4. These results suggest that antibodies with ZIKV neutralization activity also demonstrated cross‐reactivity with DENV serotypes. This finding suggests that, during a response to ZIKV protein extract, the antibodies produced can recognize shared epitopes across ZIKV and DENV serotypes. All anti‐ZIKV mAbs tested were capable of neutralizing DENV‐1. Figure 5 provides a schematic representation of DENV serotypes neutralized by each antibody. Although the mAbs showed neutralizing activity against DENV‐1, none exhibited neutralizing activity against DENV‐4. Furthermore, mAbs P62D8 and P82F6 were able to neutralize two of the four DENV serotypes, whereas P81D5 neutralized three, suggesting broader cross‐neutralization potential.

**FIGURE 3:**
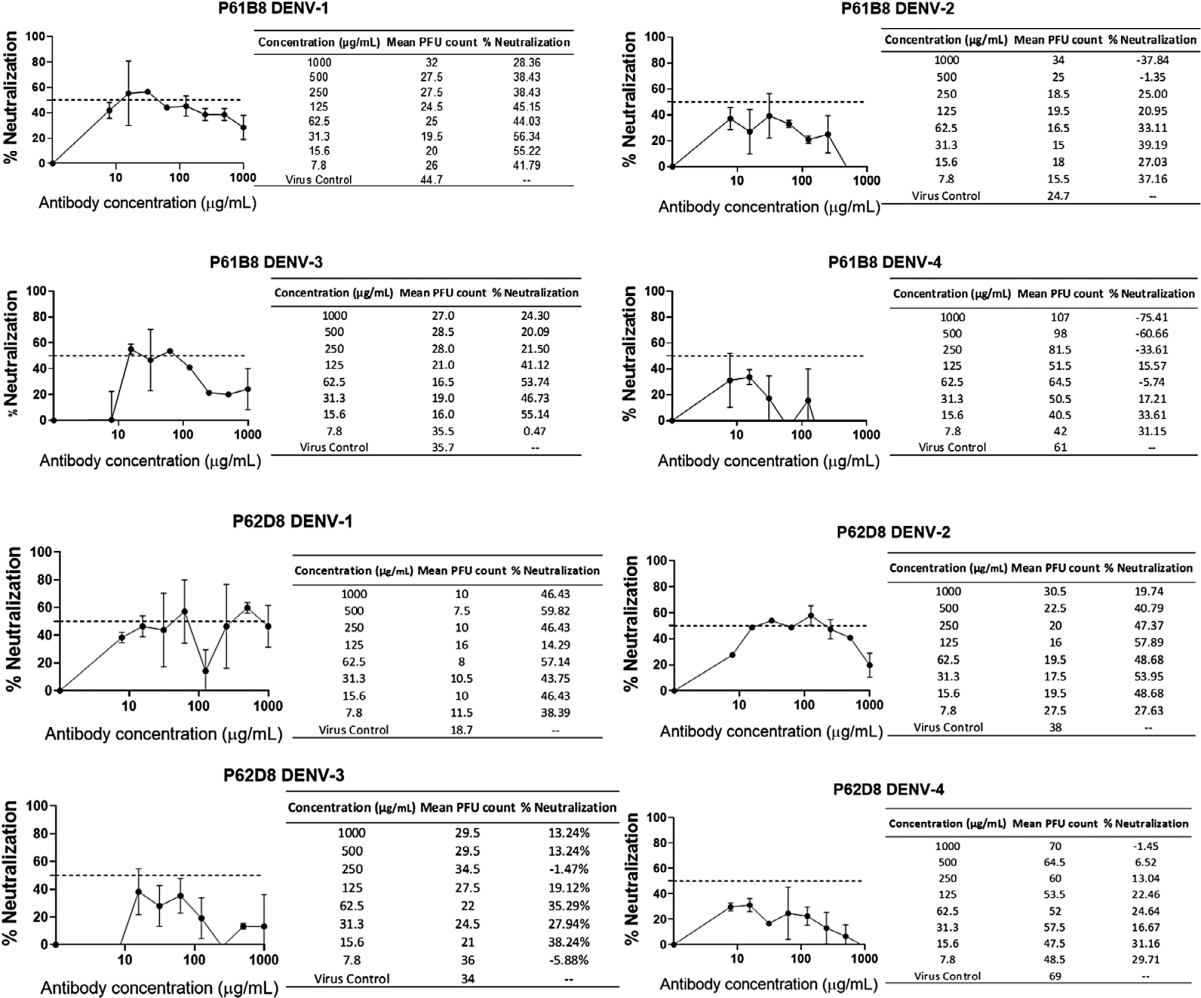
Murine monoclonal antibodies (P61B8, P62D8) produced in response to ZIKV protein extract with the ability to neutralize DENV serotypes. IC 50 P61B8 = 15.6 µg/mL (DENV‐1, and DENV‐3). IC 50 P62D8 = 62.5 µg/mL (DENV‐1); 31.3 µg/mL (DENV‐2). Data are presented as mean ± SD of duplicate measurements.

**FIGURE 4:**
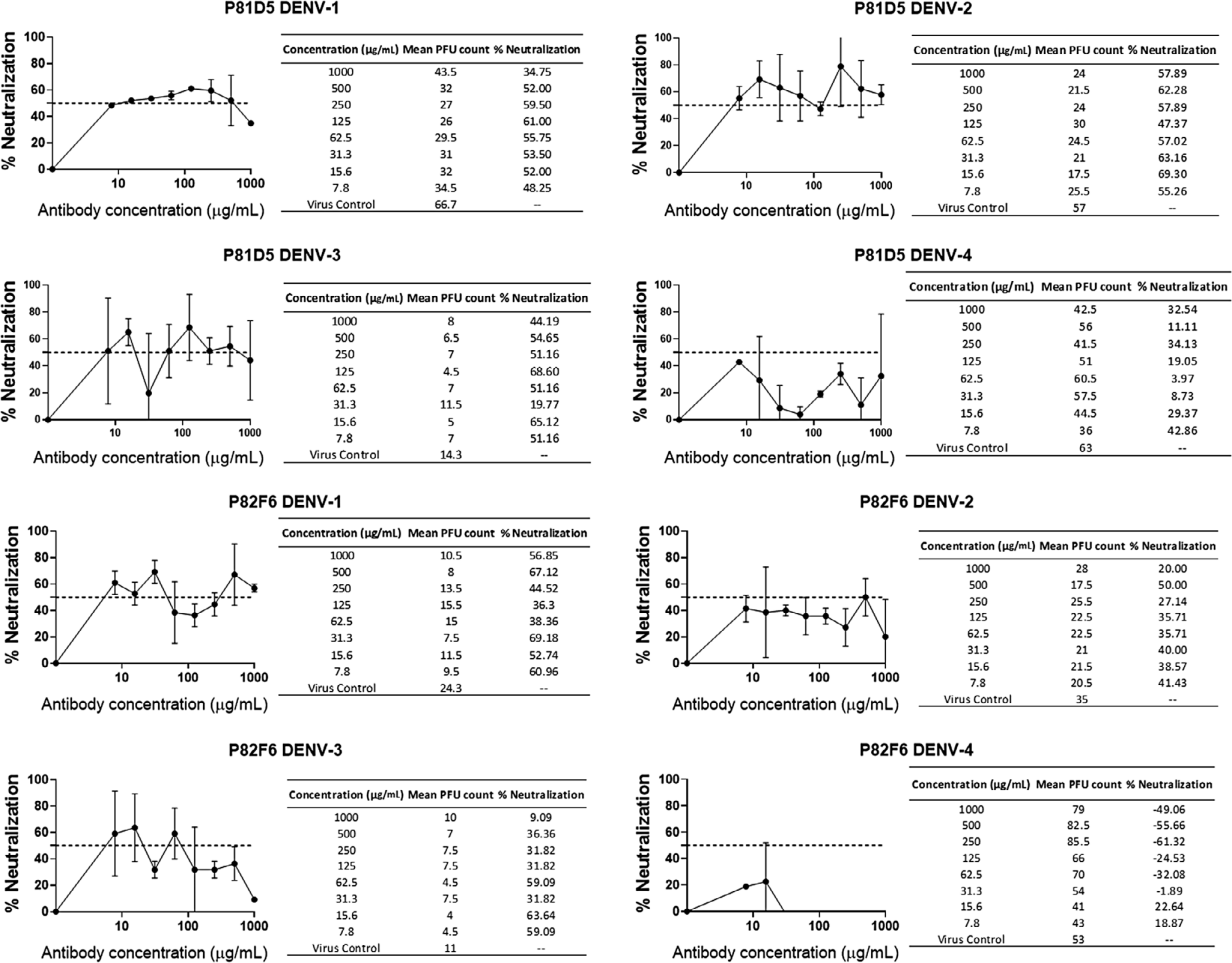
Murine monoclonal antibodies (P8D, P8F6) produced in response to ZIKV protein extract with the ability to neutralize DENV serotypes. IC 50 P81D5 = 15.6 µg/mL (DENV‐1); 7.8 µg/mL (DENV‐2 and DENV‐3). IC 50 P82F6 = 7.8 µg/mL (DENV‐1, and DENV‐3); 500 µg/mL (DENV‐2). Data are presented as mean ± SD of duplicate measurements, with exceptions.

**FIGURE 5:**
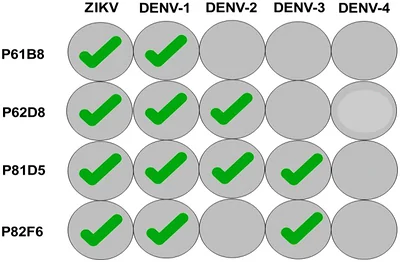
Neutralizing murine monoclonal antibodies produced in response to ZIKV protein extract with cross‐reactivity to DENV serotypes.

In this study, antibodies were tested against protein extracts from Zika and dengue viruses as well as recombinant proteins from these viruses, using Western blot analysis. The results are presented in Figures S3–S7. Protein extracts from uninfected C6/36 cells were prepared to evaluate antibody interactions with cellular constituents, as these cells were used for virus propagation (Figure S3). Poly 2, 3, 4, 5, and 7 did not recognize the ZIKV protein extract or any of the recombinant proteins tested (Figures S3–S5). Similarly, these antibodies failed to recognize DENV protein extract, with the exception of Poly 2 and Poly 5, which specifically recognized a DENV protein with an approximate molecular weight of 100–75 kDa (Figure S3).

Poly 6 and 8 detected linear epitopes in the ZIKV protein extract (100–75 kDa) but did not recognize DENV protein extracts (Figure S3). While they failed to recognize the EDIIIZK antigen (Figure S5), they successfully bound to recombinant proteins E2 and/or E6 (Figures S6 and S7). In contrast, their monoclonal derivatives—P61B8, P61C3, P62C11, P81B5, P81D5, and P82F6 – showed specificity for the E2 antigen (Figures S6 and S7). None of the tested antibodies recognized the Multi‐ZIKV, Multi‐DENV, NS1ZK, NS1D1, NS1D2, or EDIIIZK proteins (Figures S4 and S5). These findings suggest that the linear epitopes targeted by these antibodies are present in specific recombinant proteins, such as E2 and E6, but absent in the other antigens tested.

## 4 Discussion

Our findings revealed that the polyclonal antibody response induced by immunization with a ZIKV protein extract generates diverse antibodies, including those capable of discriminating between linear and conformational epitopes across ZIKV and DENV strains, as well as cross‐reactive antibodies exhibiting viral neutralization activity. Several human population studies have documented this phenomenon [15, 16, 17]. However, in regions where ZIKV and DENV co‐circulate, repeated infections with various DENV serotypes complicate the interpretation of cross‐reactivity. It remains unclear whether such cross‐reactivity arises from the activation of memory B cells from previous infections or from ZIKV‐specific antibodies that also recognize DENV epitopes. Our results suggest the latter hypothesis, as ZIKV protein extract–induced murine antibodies recognized epitopes on flavivirus proteins and effectively neutralized a specific DENV strain. A recent study showed that PEG conjugation of computationally designed ZIKV prM peptides increased cell proliferation and IFN‐γ secretion [18]. In our study, immunization with concentrated ZIKV protein extracts formulated with PEG may likewise have enhanced antigen immunogenicity.

The high structural homology between ZIKV and DENV leads to shared amino acid sequences and antigenic epitopes, resulting in a cross‐reactive immune response [19]. Primary infection with one DENV serotype confers long‐term homologous immunity, but provides only temporary or partial heterologous immunity to other serotypes. This complex phenomenon may also extend to ZIKV and DENV due to their antigenic similarity [20]. Evidence from human studies, in vitro experiments, animal models, and non‐human primates has yielded mixed results regarding whether pre‐existing immunity protects against, exacerbates, or has no effect on subsequent flavivirus infections [15, 16, 17, 21, 22, 23].

One possible explanation for these varied outcomes lies in the structural heterogeneity of ZIKV and DENV particles. Differences in glycoprotein composition and conformation at various stages of the viral life cycle, particularly in the prM and E proteins, may influence antibody recognition and neutralization. Immature viral particles with uncleaved prM are noninfectious but can promote antibody‐dependent enhancement [24]. Additionally, different regions within the E protein domains (EDI, EDII, and EDIII) are associated with varying antibody responses. While EDI and EDII are implicated in cross‐reactivity between ZIKV and DENV, EDIII tends to induce serotype‐specific responses [25].

Most potent neutralizing antibodies target conformational epitopes on the surface of flavivirus particles, but linear epitopes can also stimulate neutralizing antibody production [26]. Our results suggest that ZIKV protein extract ‐induced antibodies recognized both conformational and linear epitopes. Notably, lower concentrations of mAbs P61B8, P62D8, P81D5, and P82F6 showed neutralizing activity against specific DENV serotypes.

Antigen recognition by antibodies involves noncovalent and reversible binding. Several types of interactions contribute to antibody–antigen binding, including electrostatic forces, hydrogen bonds, Van der Waals forces, and hydrophobic interactions. When multivalent antigens are mixed with specific antibodies at appropriate concentrations, immune complexes are formed within the so‐called equivalence zone, where antigen and antibody establish an extensive network of cross‐links, thereby stabilizing the interaction. These immune complexes may subsequently dissociate either due to an increase in free antigen concentration, a phenomenon known as the antigen excess zone, or due to an increase in free antibody concentration (antibody excess zone), which can displace antibodies bound to antigenic determinants. Another factor that may interfere with the stability of immune complexes is antigen–antibody binding affinity, which is mediated by the dissociation constant (Kd). Therefore, both antibody affinity and concentration may contribute to the observed results. Importantly, the absence of surface Fcγ receptor expression on Vero cells further supports the interpretation that the negative neutralization values observed at high antibody concentrations are unlikely to result from a classical Fcγ receptor‐mediated ADE mechanism.

Understanding the impact of pre‐existing immunity on subsequent flavivirus infections is critical. While most studies focus on the effect of DENV immunity on ZIKV infection, few investigate the inverse relationship. Our findings, consistent with Watanabe et al. (2018) [27], suggest that ZIKV infection induces cross‐reactive neutralizing antibodies that target specific DENV serotypes. However, this cross‐reactivity may vary depending on the serotype involved and the dominance of neutralizing versus nonneutralizing response.

Contradictory findings, such as those reported by Larocca et al. (2021) [28], highlight the complexity of cross‐reactive immunity. They demonstrated an absence of cross‐reactive neutralizing antibodies against ZIKV induced by an attenuated DENV vaccine in murine and Rhesus monkey models. On the other hand, several studies have shown that individuals with previous dengue infections are capable of producing neutralizing antibodies for both ZIKV and DENV, with shared epitopes between these flaviviruses concentrated in the DI and DIII domains of the E protein, depending on the DENV serotype involved. These discrepancies may be explained by differences in experimental models, antigenic determinants, and immune response patterns. Our results may be aligned with studies showing that shared epitopes, particularly within the E protein DI and DIII domains, are critical for cross‐neutralization [17, 30, 31].

Previous studies demonstrated that antibodies isolated from patients infected with dengue virus were capable of neutralizing all four serotypes and also exhibited cross‐reactivity with Zika virus [32, 33, 34]. Interestingly, the broad neutralizing activity of these antibodies appears to depend not only on epitope conservation, but also on the structural and topological organization of these epitopes on the viral surface [35]. Consistent with these findings, our results showed that immunization with ZIKV protein extracts elicited a polyclonal antibody response with variable neutralizing activity against distinct DENV serotypes, suggesting that differences in epitope recognition and accessibility may influence the cross‐neutralization profile of the generated antibodies.

However, the possibility that cross‐reactive antibodies may exert concentration‐, serotype‐, and strain‐dependent effects cannot be excluded. In particular, potential antibody‐dependent enhancement (ADE) related implications were not evaluated in the present study.

## 5 Conclusions

Our study showed that immunization with a ZIKV protein extract induces the production of neutralizing antibodies that recognize epitopes shared with DENV serotypes, particularly DENV‐1. These findings suggest the presence of cross‐reactive neutralizing antibody responses between ZIKV and DENV, possibly with a serotype bias.

## Author Contributions

**Lucimeire Antonelli da Silveira**: literature search, study design, data collection, data analysis and interpretation, animal model, and writing. **Djairo Pastor Saavedra**: literature search, figures and tables, study design, data collection, data analysis and interpretation, animal model, and writing. **Isabela Cinquini Junqueira**: literature search, study design, data collection, data analysis and interpretation, animal model, and writing. **Jefferson do Carmo Dietz**: Literature search, figures and tables, study design, data collection, data analysis and interpretation, animal model, and writing. **Cristina Rodrigues de Oliveira Teixeira**: literature search, study design, data collection, data analysis and interpretation, animal model, and writing. **Leonardo Lopes‐ Luz**: literature search, study design, data collection, data analysis and interpretation, animal model, and writing. **Lorena Carvalho de Souza Chaves**: literature search, study design, data collection, data analysis and interpretation, animal model, and writing. **Bergmann Morais Ribeiro**: study design, data collection, data analysis and interpretation, animal model, and writing. All authors read and approved the final manuscript.

## Funding

This research was funded by the Foundation for Research Support of the State of Goiás (FAPEG—Public Call #04/2017).

## Ethics Statement

All animal experiments were carried out according to the guidelines outlined by the National Council for the Control of Animal Experimentation (CONCEA). The animal protocol was approved by the Ethics Committee on Animal Use (CEUA) of the Federal University of Goiás (CEUA permit no. 101/15).

## Conflicts of Interest

The authors declare no conflicts of interest.

## Supporting information




**Supporting File 1**: biot70323‐sup‐0001‐SuppMat.docx.

**Supporting File 2**: biot70323‐sup‐0002‐FiguraS1.pdf.

**Supporting File 3**: biot70323‐sup‐0003‐FiguraS2.pdf.

**Supporting File 4**: biot70323‐sup‐0004‐FigureS3.docx.

**Supporting File 5**: biot70323‐sup‐0005‐FigureS4.docx.

**Supporting File 6**: biot70323‐sup‐0006‐FigureS5.docx.

**Supporting File 7**: biot70323‐sup‐0007‐FigureS6.docx.

**Supporting File 8**: biot70323‐sup‐0008‐FigureS7.docx.

## Data Availability

The datasets used and analyzed in the current study are available from the corresponding author upon reasonable request.
