# Supplementary material for: Cross‐Reactive Neutralizing Antibodies Between ZIKV and DENV May Exhibit Serotype Specificity
Source: Biotechnol J. 2026 Oct 26;21(10):e70323. doi: 10.1002/biot.70323 (PMC13696971; doi:10.1002/biot.70323)
Supplement: Supplementary file 1 [file BIOT-21-e70323-s001.pdf]

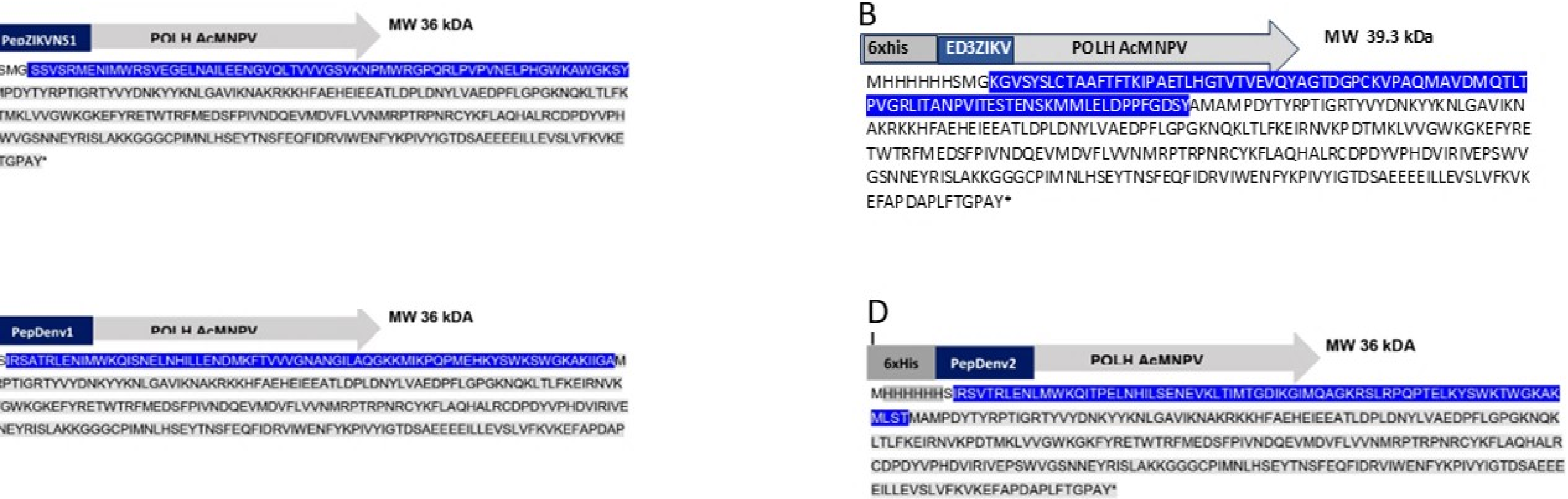

Recombinant DENV and ZIKV fused proteins. Four recombinant proteins were expressed in insect cells as sing baculovírus vetors. An immunogenic peptide rigion of the NS1 (A) and Envelope (B) proteins from Z ogenic peptides from DENV-1 (C) and DENV-2 (D), were fused to the polyhedrin protein of the baculovíru na californica multiple nucleopolyhedrovirus (AcMNPV) and expressed in insect cell. All fused proteins cor ines tag at the N-terminal region. The molecular weight of each protein is indeicated.
