# Supplementary material for: Cross‐Reactive Neutralizing Antibodies Between ZIKV and DENV May Exhibit Serotype Specificity
Source: Biotechnol J. 2026 Oct 26;21(10):e70323. doi: 10.1002/biot.70323 (PMC13696971; doi:10.1002/biot.70323)
Supplement: Supplementary file 2 [file BIOT-21-e70323-s004.pdf]

A. E6 (preM-E) 76.52 kDa

RRGADTSVGIVGLLLTTAMAAEVTRRGSAYYMYLDRNDAGEAISFPTTLGMNKCYIQIMDLGHMCDATMSYECPMLDEGVEPDD
DCWCNTTSTWVVYGTCHHKKGEARRSRRAVTLPSHSTRKLQTRSQTWLESREYTKHLIRVENWIFRNPGFALAAAAIAWLLGSSTS
KVIYLVMILLIAPAYSIRCIGVSNRDFVEGMSGGTWVDVVLEHGGCVTVMAQDKPTVDIELVTTTVSNMAEVRSYCYEASISDMAS
SRCPTQGEAYLDKQSDTQYVCKRTLVDRGWGNGCGLFGKGSLVTCAKFACSKKMTGKSIQPENLEYRIMLSVHGSQHSGMIVNDT
HETDENRAKVEITPNSPRAEATLGGFGSLGLDCEPRTGLDFSDLYYLTMNNKHWLVHKEWFHDIPLPWHAGADTGTPHWNNKEA
EFKDAHAKRQTVVVLGSQEGAVHTALAGALEAEMDGAKGRLSSGHLKCRLKMDKLRLKGVSYSLCTAAFTFTKIPAETLHGTVTVE
QYAGTDGPCKVPAQMAVDMQTLTPVGRLITANPVITESTENSKMMLELDPPFGDSYIVIGVGEKKITHHWHRSGSTIGKAFEATVR
AKRMAVLGDTAWDFGSVGGALNSLGKGIHQIFGAAFKSLFGGMSWFSQILIGTLLMWLGLNTKNGSISLMCLALGGVLIFLSTAVSA
HHHHHH

. E2 (C-preM-E) 87.71 kDa

KNPKKKSGGFRIVNMLKRGVARVSPFGGLKRLPAGLLLGHGPIRMVLAILAFLRFTAIKPSLGLINRWGSVGKKEAMEIIKKFKKDLA
MLRIINARKEKKRRGADTSVGIVGLLLTTAMAAEVTRRGSAYYMYLDRNDAGEAISFPTTLGMNKCYIQIMDLGHMCDATMSYEC
MLDEGVEPDDVDCWCNTTSTWVVYGTCHHKKGEARRSRRAVTLPSHSTRKLQTRSQTWLESREYTKHLIRVENWIFRNPGFALAA
IAWLLGSSTSQKVIYLVMILLIAPAYSIRCIGVSNRDFVEGMSGGTWVDVVLEHGGCVTVMAQDKPTVDIELVTTTVSNMAEVRS
YEASISDMASDSRCPTQGEAYLDKQSDTQYVCKRTLVDRGWGNGCGLFGKGSLVTCAKFACSKKMTGKSIQPENLEYRIMLSVHG
HSGMIVNDTGHETDENRAKVEITPNSPRAEATLGGFGSLGLDCEPRTGLDFSDLYYLTMNNKHWLVHKEWFHDIPLPWHAGAD
TPHWNNKEALVEFKDAHAKRQTVVVLGSQEGAVHTALAGALEAEMDGAKGRLSSGHLKCRLKMDKLRLKGVSYSLCTAAFTFTKI
ETLHGTVTVEVQYAGTDGPCKVPAQMAVDMQTLTPVGRLITANPVITESTENSKMMLELDPPFGDSYIVIGVGEKKITHHWHRSG
GKAFEATVRGAKRMAVLGDTAWDFGSVGGALNSLGKGIHQIFGAAFKSLFGGMSWFSQILIGTLLMWLGLNTKNGSISLMCLAL
VLIFLSTAVSADHHHHHH

Recombinant structural poliproteins of ZIKV. Two versions of the estructural poliproteins of ZIKV were exp
s using baculovirus vectors. The full-length structural protein E2 containing the c-preM and E proteins (A)
version with the pre-M and E proteins (B). The 102 amino acids difference between the two sequences a
he two recombinat proteins containined a six-histindines tag at the C-terminal region. The molecular wei
indicated.
