# Supplementary material for: Cross‐Reactive Neutralizing Antibodies Between ZIKV and DENV May Exhibit Serotype Specificity
Source: Biotechnol J. 2026 Oct 26;21(10):e70323. doi: 10.1002/biot.70323 (PMC13696971; doi:10.1002/biot.70323)
Supplement: Supplementary file 3 [file BIOT-21-e70323-s002.docx]

**Figure S3 –** Western Blot with anti-Zika antibodies against protein extracts from flavivirus and C6/36 cells. MW - Molecular weight marker. (A) Poly 2 antibodies against protein extracts of DENV, ZIKV, and C6/36 cells. (B) Poly 4 antibodies against protein extracts of DENV, ZIKV, and C6/36 cells. (C) Poly 5 antibodies against protein extracts of DENV, ZIKV, and C6/36 cells. (D) Poly 6 antibodies against protein extracts of DENV, ZIKV, and C6/36 cells. (E) Poly 8 antibodies against protein extracts of DENV and ZIKV.
