# Supplementary material for: Cross‐Reactive Neutralizing Antibodies Between ZIKV and DENV May Exhibit Serotype Specificity
Source: Biotechnol J. 2026 Oct 26;21(10):e70323. doi: 10.1002/biot.70323 (PMC13696971; doi:10.1002/biot.70323)
Supplement: Supplementary file 4 [file BIOT-21-e70323-s003.docx]

**Figure S7 –** Western Blot with Poly 8 and mAbs against ZIKV protein extract and ZIKV recombinant proteins. E2 - ZIKV Structural Protein 2 (SS, Pre-M, M, and E). E6 - ZIKV Structural Protein 6 (Cap, SS, Pre-M, M, and E).. (A) Poly 8 against ZIKV protein extract and recombinant antigens E2 and E6. (B) mAb P81B5 against ZIKV protein extract and recombinant antigens E2 and E6. (C) mAb P81D5 against ZIKV protein extract and recombinant antigens E2 and E6. (D) mAb P81H6 against ZIKV protein extract and recombinant antigens E2 and E6. (E) mAb P82F6 against ZIKV protein extract and recombinant antigens E2 and E6.
