# Supplementary material for: Cross‐Reactive Neutralizing Antibodies Between ZIKV and DENV May Exhibit Serotype Specificity
Source: Biotechnol J. 2026 Oct 26;21(10):e70323. doi: 10.1002/biot.70323 (PMC13696971; doi:10.1002/biot.70323)
Supplement: Supplementary file 5 [file BIOT-21-e70323-s005.docx]

**Figure S6 –** Western Blot with Poly 6 and mAbs against ZIKV protein extract and ZIKV recombinant proteins. E2 - ZIKV Structural Protein 2 (SS, Pre-M, M, and E). E6 - ZIKV Structural Protein 6 (Cap, SS, Pre-M, M, and E). (A) Poly 6 against ZIKV protein extract and recombinant antigens E2 and E6. (B) mAb P61B8 against ZIKV protein extract and recombinant antigens E2 and E6. (C) mAb P61C3 against ZIKV protein extract and recombinant antigens E2 and E6. (D) mAb P62C11 against ZIKV protein extract and recombinant antigens E2 and E6. (E) mAb P62D8 against ZIKV protein extract and recombinant antigens E2 and E6.
