# Supplementary material for: Cross‐Reactive Neutralizing Antibodies Between ZIKV and DENV May Exhibit Serotype Specificity
Source: Biotechnol J. 2026 Oct 26;21(10):e70323. doi: 10.1002/biot.70323 (PMC13696971; doi:10.1002/biot.70323)
Supplement: Supplementary file 6 [file BIOT-21-e70323-s006.docx]

**Figure S5 –** Western Blot with anti-Zika polyclonal antibodies against recombinant flavivirus proteins. NS1D1 - Non-Structural Protein 1 of DENV-1. NS1D2 - Non-Structural Protein 1 of DENV-2. NS1ZK) - Non-Structural Protein 1 of ZIKV. EDIIIZK - Domain III of ZIKV E Protein. MW – Molecular weight marker. (A) Poly 3 against recombinant antigens NS1D1, NS1D2, NS1ZK, and EDIIIZK. (B) Poly 7 against recombinant antigens NS1D1, NS1D2, NS1ZK, and EDIIIZK. (C) Poly 8 against recombinant antigens NS1D1, NS1D2, NS1ZK, and EDIIIZK.
