# Supplementary material for: Cross‐Reactive Neutralizing Antibodies Between ZIKV and DENV May Exhibit Serotype Specificity
Source: Biotechnol J. 2026 Oct 26;21(10):e70323. doi: 10.1002/biot.70323 (PMC13696971; doi:10.1002/biot.70323)
Supplement: Supplementary file 7 [file BIOT-21-e70323-s007.docx]

**Figure S4 –** Western Blot with anti-Zika polyclonal antibodies against protein extracts from flavivirus and recombinant flavivirus proteins. MW - Molecular weight marker. (A) Control of Multi DENV and Multi ZIKV antigens tested with anti-His antibodies. (B) Poly 3 against protein extracts of DENV and ZIKV and recombinant antigens Multi DENV and Multi ZIKV. (C) Poly 7 against protein extracts of DENV and ZIKV and recombinant antigens Multi DENV and Multi ZIKV. (D) Poly 8 against protein extracts of DENV and ZIKV and recombinant antigens Multi DENV and Multi ZIKV
