# Supplementary material for: Cross‐Reactive Neutralizing Antibodies Between ZIKV and DENV May Exhibit Serotype Specificity
Source: Biotechnol J. 2026 Oct 26;21(10):e70323. doi: 10.1002/biot.70323 (PMC13696971; doi:10.1002/biot.70323)
Supplement: Supplementary file 8 [file BIOT-21-e70323-s008.docx]

**Supplementary information**

**Figure S5 –** Western Blot with anti-Zika antibodies against protein extracts from flavivirus and C6/36 cells. MW - Molecular weight marker. (A) Poly 2 antibodies against protein extracts of DENV, ZIKV, and C6/36 cells. (B) Poly 4 antibodies against protein extracts of DENV, ZIKV, and C6/36 cells. (C) Poly 5 antibodies against protein extracts of DENV, ZIKV, and C6/36 cells. (D) Poly 6 antibodies against protein extracts of DENV, ZIKV, and C6/36 cells. (E) Poly 8 antibodies against protein extracts of DENV and ZIKV.

**Figure S6 –** Western Blot with anti-Zika polyclonal antibodies against protein extracts from flavivirus and recombinant flavivirus proteins. MW - Molecular weight marker. (A) Control of Multi DENV and Multi ZIKV antigens tested with anti-His antibodies. (B) Poly 3 against protein extracts of DENV and ZIKV and recombinant antigens Multi DENV and Multi ZIKV. (C) Poly 7 against protein extracts of DENV and ZIKV and recombinant antigens Multi DENV and Multi ZIKV. (D) Poly 8 against protein extracts of DENV and ZIKV and recombinant antigens Multi DENV and Multi ZIKV.

**Figure S7 –** Western Blot with anti-Zika polyclonal antibodies against recombinant flavivirus proteins. NS1D1 - Non-Structural Protein 1 of DENV-1. NS1D2 - Non-Structural Protein 1 of DENV-2. NS1ZK) - Non-Structural Protein 1 of ZIKV. EDIIIZK - Domain III of ZIKV E Protein. MW – Molecular weight marker. (A) Poly 3 against recombinant antigens NS1D1, NS1D2, NS1ZK, and EDIIIZK. (B) Poly 7 against recombinant antigens NS1D1, NS1D2, NS1ZK, and EDIIIZK. (C) Poly 8 against recombinant antigens NS1D1, NS1D2, NS1ZK, and EDIIIZK.

**Figure S8 –** Western Blot with Poly 6 and mAbs against ZIKV protein extract and ZIKV recombinant proteins. E2 - ZIKV Structural Protein 2 (SS, Pre-M, M, and E). E6 - ZIKV Structural Protein 6 (Cap, SS, Pre-M, M, and E). (A) Poly 6 against ZIKV protein extract and recombinant antigens E2 and E6. (B) mAb P61B8 against ZIKV protein extract and recombinant antigens E2 and E6. (C) mAb P61C3 against ZIKV protein extract and recombinant antigens E2 and E6. (D) mAb P62C11 against ZIKV protein extract and recombinant antigens E2 and E6. (E) mAb P62D8 against ZIKV protein extract and recombinant antigens E2 and E6.

**Figure S9 –** Western Blot with Poly 8 and mAbs against ZIKV protein extract and ZIKV recombinant proteins. E2 - ZIKV Structural Protein 2 (SS, Pre-M, M, and E). E6 - ZIKV Structural Protein 6 (Cap, SS, Pre-M, M, and E).. (A) Poly 8 against ZIKV protein extract and recombinant antigens E2 and E6. (B) mAb P81B5 against ZIKV protein extract and recombinant antigens E2 and E6. (C) mAb P81D5 against ZIKV protein extract and recombinant antigens E2 and E6. (D) mAb P81H6 against ZIKV protein extract and recombinant antigens E2 and E6. (E) mAb P82F6 against ZIKV protein extract and recombinant antigens E2 and E6.
